# Network analysis of post-traumatic stress disorder symptoms in stroke patients

**DOI:** 10.3389/fpsyt.2025.1663366

**Published:** 2025-09-18

**Authors:** Yingying Li, Yanchun Li, Zhenmei Zhang

**Affiliations:** ^1^ School of Medicine, Qilu Institute of Technology, Jinan, Shandong, China; ^2^ School of Nursing, Qilu Medical University, Zibo, Shandong, China; ^3^ Department of Nursing, Shandong Provincial Hospital Affiliated to Shandong First Medical University; Shandong Provincial Hospital, Shandong University, Jinan, Shandong, China

**Keywords:** PTSD, stroke, network analysis, post-traumatic stress disorder, symptoms

## Abstract

**Background:**

Stroke patients have a high incidence of Post-traumatic stress disorder (PTSD). Previous studies on PTSD in stroke patients mainly focus on the risk factors and possible harms caused by PTSD and use the overall score to explain the severity of PTSD. The interconnections and effects of symptoms are ignored. Network analysis is a statistical method that can discover and visualize complex relationships between multiple variables. The purpose of this study was to identify the central and core symptoms in the symptom network of PTSD in stroke patients.

**Methods:**

315 patients diagnosed with cerebral apoplexy were selected as the study objects. Symptoms of PTSD were assessed using the Event Impact Scale (IES-R). The graph Gaussian model is used to estimate the network model. To clarify the network relationship and core symptoms of PTSD in stroke patients. The network’s stability and accuracy are tested using the discard example method and non-parametric bootstrap method.

**Result:**

The network analysis found that A11 (I tried not to think about it) has the most substantial relationship with I3 (Other things kept making me think about it). I6 (I thought about it when I didn’t mean to) has the most substantial relationship with I9 (Pictures about it popped into my mind). “I was jumpy and easily startled”(H10) is the core symptom of PTSD in stroke patients. The network structure is suitable for stability and accuracy tests.

**Conclusion:**

It is possible to reduce the severity of PTSD in stroke patients and promote their personal growth by taking timely intervention measures according to the identified central symptoms of PTSD.

## Introduction

Stroke is the leading cause of death worldwide (1), accounting for 11.8% of deaths worldwide (2), and is the leading cause of long-term physical, cognitive or psychological disability. Psychological complications of stroke include depression and anxiety in 29-33% of stroke survivors (3–5). Due to the sudden onset of stroke, it is difficult to predict and uncontrollable, which seriously affects the physical and mental health of patients and causes post-traumatic stress disorder (PTSD) (6). PTSD is a form of mental trauma that is delayed or persists for a long time after an individual experiences sudden, threatening, or even catastrophic events (7). The main manifestations are that the content or scenes related to trauma repeatedly surface in the mind uncontrollably. Individuals often resist participating in activities related to the traumatic event and try to avoid places and people related to the traumatic event (7). In addition, patients may also experience emotional reactions such as irritability and excessive alertness. Some people may even show somatization symptoms of anxiety (8). The prevalence rate of PTSD after stroke is up to 18% (9). PTSD not only affects patients’ interpersonal communication, work, and life but also comorbidities such as anxiety, depression, drug abuse, and fear, which seriously affect patients’ quality of life and mental health. Research shows that most people with PTSD are not detected and treated in time and that this traumatic event causes widespread suffering for almost everyone (10, 11). Foreign studies have shown that PTSD significantly affects patients’ cognitive problems and adaptation problems (12), and the more severe the PTSD symptoms, the worse the cognitive function (13).There are various clinical symptoms of PTSD, and the interaction between symptoms will affect the progression of the disease (14). Previous studies on PTSD in stroke patients mainly focus on the risk factors of PTSD and the possible harm caused by PTSD, using the overall score. To explain the severity of PTSD, it is assumed that each symptom or item is of equal importance, ignoring the correlation and function of the symptoms (15).

Borsboom proposed the network theory of mental disorder (NTMD) in 2017 (16). This theory holds that mental disorders are complex network systems formed by the interactions among symptoms, and the direct interactions among symptoms lead to the emergence of mental disorders (16). Specifically, a certain stimulus may activate a certain symptom, and the emergence of this symptom will further activate other related symptoms (17). Thus, a continuous activation cycle is formed. When the activated symptom cluster reaches a certain diagnostic criterion, mental disorders occur. Network analysis is a data analysis method based on network theory (17). It is a statistical approach capable of discovering and visualizing complex relationships among multiple variables and identifying potential influencing factors (18). An essential goal of network analysis methods is identifying central and bridging symptoms in a network and analyzing the mechanisms affecting its connectivity (19). A network diagram visually represents a system consisting of nodes and edges. Nodes represent variables, and edges represent connections between nodes. The centrality index is used to identify the most critical nodes in the network, including strength, tightness, and intermediateness (20). Strength centrality determines the strength of the relationship between a node and its neighbors. Proximity centrality represents the average distance between a node and other nodes. Intermediate centrality represents the frequency at which a node is connected between two nodes (21). Researchers can better understand the mechanisms underpinning component-to-symptom interaction and provide more accurate intervention targets at a finer level with the use of NA (22).

The central symptoms vary from person to person. Therefore, based on NA, this study attaches importance to the interaction of PTSD symptoms in stroke patients and constructs PTSD symptom networks through symptom network analysis. Driven by data, this study presents the connection between symptoms and symptom interactions through network maps, intuitively identifies network features and core symptoms, and proposes corresponding nursing countermeasures. It provides the basis for exploring the target of psychological rehabilitation intervention and accurate symptom management.

## Measurement

### Participants

This is a cross-sectional study conducted from May 2024 to May 2025 in Shandong Province, China. Clinical staff recruited participants on site, and after obtaining informed consent from eligible individuals, they quickly distributed paper versions of the questionnaire. Participants were selected based on the following criteria: (1) they were at least 18 years of age; (2) they had been diagnosed as stroke by a clinician and confirmed as stroke by neuroimaging tests such as CT or MRI; (3) Clear consciousness, able to cooperate with research; (4) Express willingness to participate in this study. Exclusion criteria: (1) a history of significant anxiety, depression, mental retardation or other serious mental illness; (2) Patients with various serious physical diseases or malignant tumors; (3) recent use of antidepressants or sedatives; (4) Experienced heavy family changes or trauma in the past six months. Ultimately, 350 stroke patients participated in the study. A total of 315 people successfully completed the questionnaire, with an effective response rate of 90%.

### Sociodemographic characteristics

Participants self-reported their information, providing details of age, educational background, marital status, whether they were co-diabetics, whether they were co-hypertensive, etc.

### PTSD symptoms

Weiss and Marmar (23) revised Horowitz’s Impact of Events Scale in 1979 (24), a total of 22 items. Domestic scholars such as Guo (25) divided the scale into three factors, arousal, escape, and intrusion, according to the original text, and revised the scale. The severity of PTSD was evaluated according to the total score. When the total score was 0-8, it was subclinical without PTSD. A score of 9 to 25 is mild PTSD, 26 to 43 is moderate PTSD, and 44 to 88 is severe PTSD. The IES-R has been widely used among Chinese populations (26, 27), demonstrating good reliability and validity in this study. The Cronbach’s α coefficient of this scale was 0.95 in the study.

### Data analysis

First, the data were analyzed in the SPSS 26.0 package program. We conducted a descriptive analysis to analyze the characteristics of the participants (continuous variables: means and standard deviations (SD); categorical variables: frequencies and percentages). We computed polychoric correlations between all nodes to examine the edges of the network. Polychoric, EBIC and glasso jointly calculated the edge weight. Then, we estimated the Graphical Gaussian Model (GGM) using the graphical least absolute shrinkage and selection operator (LASSO) in combination with the Extended Bayesian Information Criterion (EBIC) model (20). In the network model, each symptom is represented as a “node” and the association between symptoms is defined as an “edge.” Nodes with high EI values can activate other nodes within the network, making them essential components of their own network (28). To identify bridge nodes that connect PTSD, we calculated the bridge expected influence (BEI). A higher positive value of the BEI for a node indicates a greater activation capacity toward nodes in another cluster. In contrast, a higher negative value signifies a more substantial deactivation capacity toward nodes in another cluster (28, 29). Bootstrapping approaches were adopted through the R package botnet to guarantee the networks’ accuracy and stability. We applied non-parametric bootstrapping (with 2,000 samples) to estimate the 95% confidence interval for all edges within the network to verify the accuracy of edge weights (20). To ensure the stability of BEI centrality, the correlation stability (CS) coefficient was obtained by a case-dropping subset bootstrap method (with 2,000 bootstrap samples) (20). It is recommended that the CS-coefficient is preferably above 0.5 and should not fall below 0.25 (20). Bootstrap difference tests (2,000 samples) were conducted to determine if there are significant differences between edge weights or between node BEIs. Bootstrap difference testing (2,000 samples) was run to determine whether significant differences exist between edge weights or node BEIs (20).

## Results

### Characteristics of the participants

A total of 315 stroke participants were investigated, of which 174 (55.2%) were male stroke participants and 141 (44.8%) were female. In terms of comorbidities, 86 (27.3%) stroke participants had diabetes, and 144 (45.7%) stroke participants had hypertension. Further demographic information on the participants is shown in **Table 1**. The total score of PTSD was 32.05 ± 15.472. The mean and standard deviation of the PTSD program are shown in **Supplementary Table S1**.

**Table 1:** Demographic characteristics of the participants (n=315).

Variable	N (%)
Gender	
Male	174(55.2)
Female	141(44.8)
Marital status	
Single	31(9.8)
Married	253(80.3)
divorced/widow	31(9.8)
Age (years)	
<60	81(25.7)
61-70	108(34.3)
71-80	103(32.7)
≥81	23(7.3)
Education	
Junior high school or less	237(75.2)
Senior middle school	48(15.2)
College	30(9.5)
Diabetes	
No	229(72.7)
Yes	86(27.3)
Hypertension	
No	171(54.3)
Yes	144(45.7)

### Network structure

**Figure 1:**
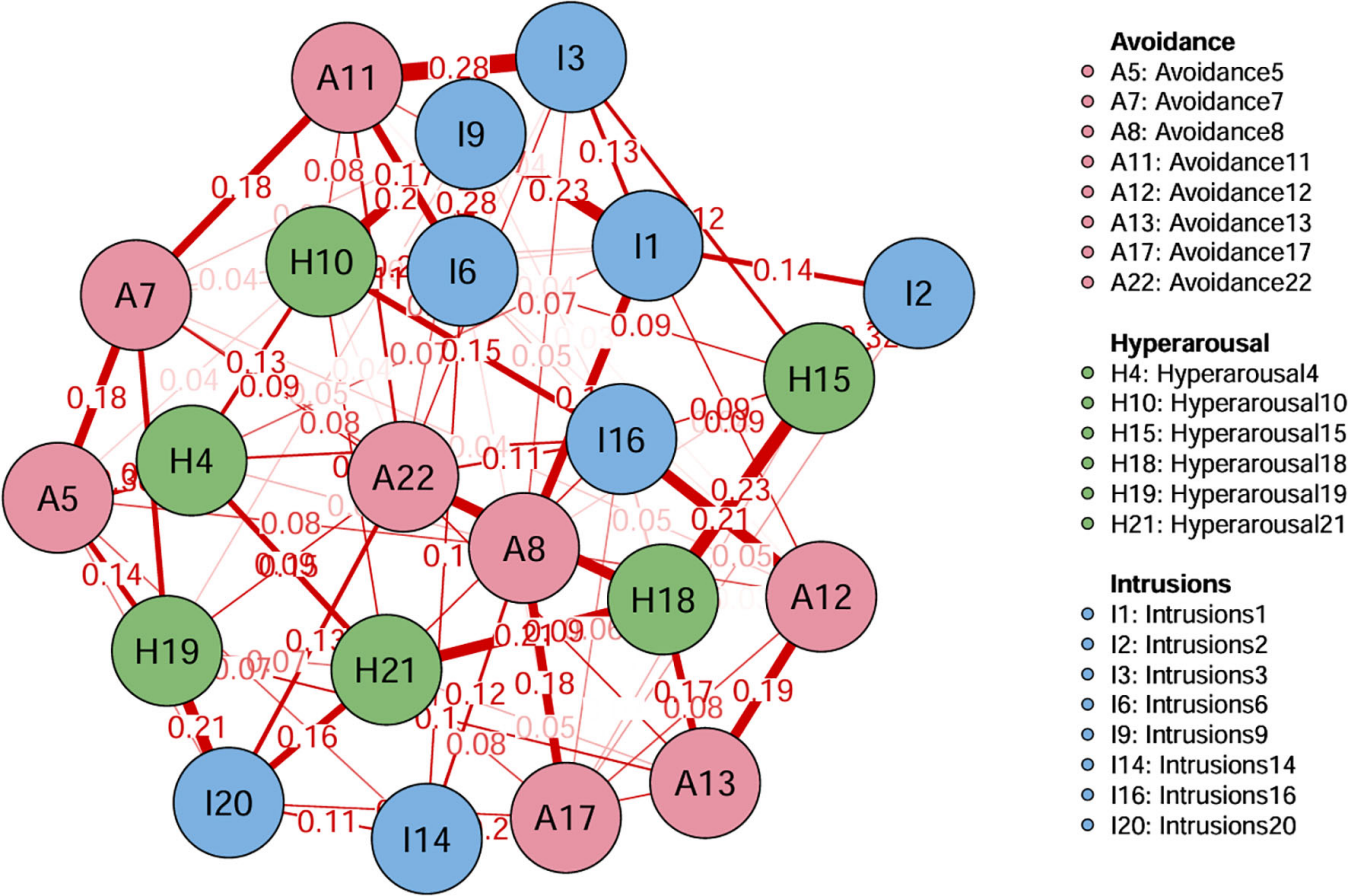
The network structure of PTSD symptoms.

**Figure 1** shows the network structure of PTSD. Two hundred thirty-one edge numbers were constructed in the whole network, and 22 symptoms in 3 dimensions were included in the entire network. Among them, the two groups with the most significant edge weight, A11 (I tried not to think about it) and I3 (Other things kept making me think about it), have an edge weight value of 0.28. I6 (I thought about it when I didn’t mean to) and I9 (Pictures about it popped into my mind) have an edge weight of 0.28. The correlation matrix for PTSD items is presented in **Supplementary Table S2**.

**Figure 2:**
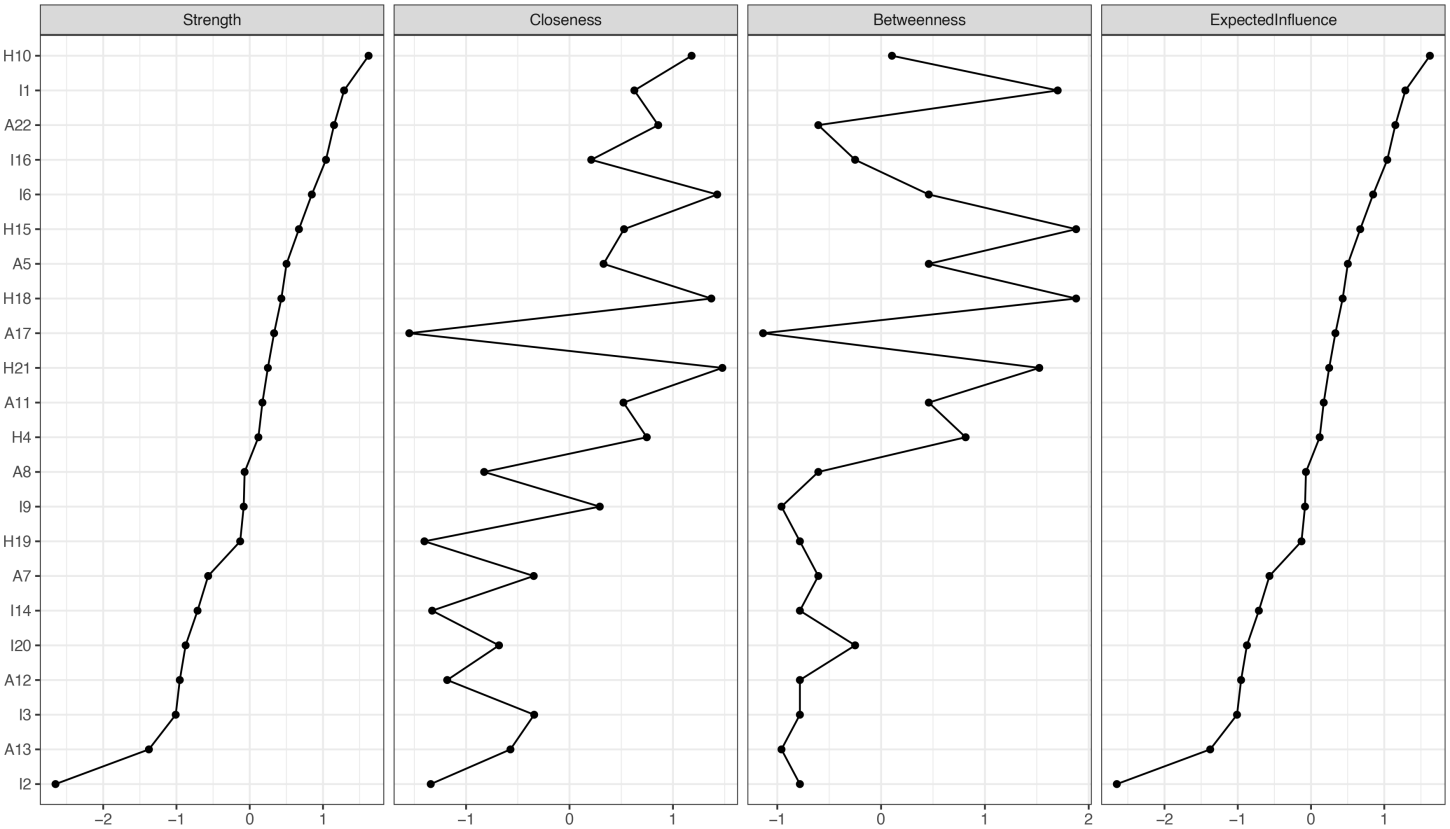
Expected influence, strength, closeness and betweenness of PTSD.

**Figure 3:**
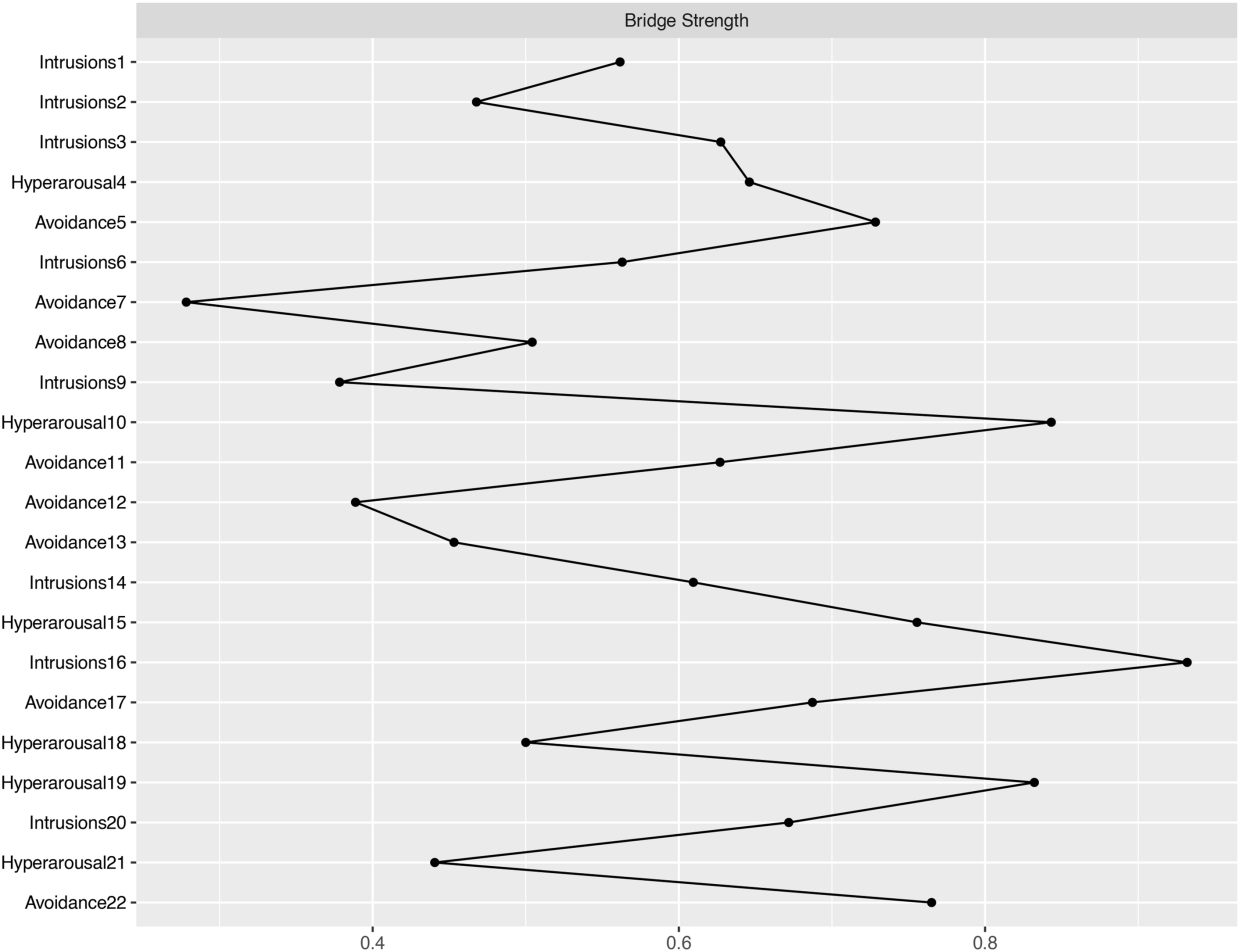
Bridge strength of PTSD.

In terms of EI centrality, network node H10 (“I was jumpy and easily startled”) has the highest EI value (1.622). This is followed by I1 (“Any reminder brought back feelings about it”) (EI value 1.288) and A22 (“ I tried not to talk about it”) (EI value 1.151). The PTSD values for each node are illustrated in **Figures 2**, **3**. The bootstrapped 95% CIs for most edge weights were relatively narrow, indicating an accurate network structure (see **Supplementary Figure S1**). In addition, the non-parametric bootstrap difference test showed significant differences between partial edge weights and node EIS (see **Supplementary Figures S2, S3**).

### Network stability and accuracy

**Figure 4:**
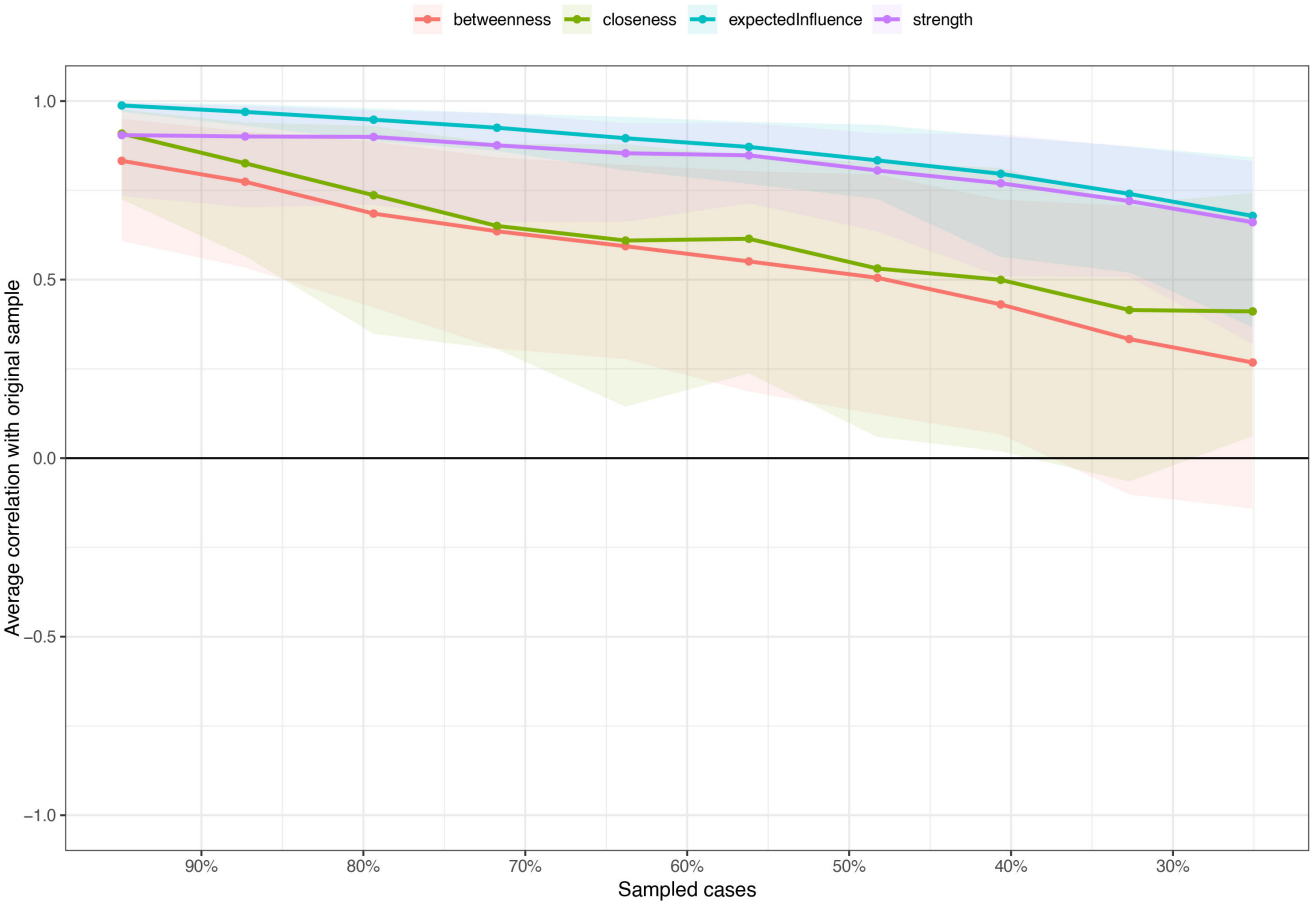
Stability of expected influence indices using case-dropping bootstrap.

Network stability and accuracy **Figure 4** shows the results of the case-dropping bootstrap test. In terms of network stability, the CS coefficient of the centrality EI is 0.517. The centrality index will change significantly only after the network samples decrease by 51.7%. Additionally, nonparametric bootstrapped difference tests revealed significant differences among most edge weights and node EIs (see **Supplementary Figures S2**, **Supplementary Figures S3**).

## Discussion

In our study, we found that the PTSD score of stroke patients was 32.05 (SD 15.472), which was at a moderate level. Stroke patients with sudden onset and severe disease, easy to cause the body’s nervous, endocrine and immune system dysfunction. The suddenness, unpredictability, uncontrollability and severity of stroke have caused severe blows to patients both physically and psychologically, which can easily lead to PTSD in patients. We suggest conducting PTSD assessment for stroke patients at an early stage, promptly formulating and implementing care plans. Additionally, through interdisciplinary collaboration, integrating professional knowledge from fields such as psychology, neuroscience, and rehabilitation medicine, we aim to further promote research and practice on PTSD in stroke patients, thereby facilitating their mental health and overall recovery.

Symptom network analysis results of this study showed that symptoms “I tried not to think about it (A11)” and symptoms “Other things kept making me think about it (I3)”, The strongest association was found between symptoms such as “I thought about it when I didn’t mean to (I6)” and “Pictures about it popped into my mind” (I9). Avoidance is a common behavior in mental illness (30). People who experience trauma often avoid thoughts, feelings, and places that remind them of the traumatic experience. In fact, individuals who have experienced trauma often distort attribution about themselves and the world, forming negative cognitive styles (31, 32). In order to avoid having this negative experience, they will shift their attention away from the thoughts and feelings associated with the traumatic event, i.e. show symptoms of thoughts avoidance. Recalling the thoughts and feelings of stroke may cause pain to patients. In order to reduce the pain, some patients will choose to avoid recalling the thoughts and feelings related to stroke experience (33), such as the feeling of numbness and dizziness during stroke attack, and the dialogue and scenes during stroke attack will also remind patients of stroke. Stroke is a sudden, significant physical and psychological traumatic event. When a patient experiences an episode of stroke, it is often accompanied by intense physical pain, fear, confusion, or a sense of death. Emotionally intense events tend to leave deep memory traces in the brain, especially those that are physically or psychologically extremely upsetting or frightening. These memories are reinforced and stored by the brain’s amygdala (emotional processing center), causing the patient’s mind to repeatedly reproduce images, situations or fragments associated with stroke. Active psychological care should be given to patients with cerebral stroke to guide them in understanding stroke and coping with trauma. Nurses should achieve precise positioning in clinical practice, help patients understand the nature of stroke and the rehabilitation process, and alleviate patients’ mental stress through measures such as cognitive therapy (34). On the basis of routine care, psychological care should be provided, and patients should be guided to correctly deal with trauma. In future research, the above symptoms should be closely monitored, and symptoms with strong preventive identification should be identified. Taking this as the entry point, a combination of early psychological intervention and drug treatment is adopted to weaken the interaction between symptoms (35), cut off the strong connection between symptoms, improve the efficiency and effect of intervention (35), and control the progression of PTSD.

Among the symptoms, “I was jumpy and easily startled”(H10) showed the highest EI value. This is an effective target for the prevention, treatment and intervention of PTSD in stroke patients. Individuals who experience trauma become more sensitive to similar stimuli or triggering events, and with a heightened panic response, the patient exhibits a heightened state of alertness (36). Studies have found that patients with PTSD have apparent abnormal changes in brain function in the amygdala, ventromedial prefrontal cortex, dorsal anterior cingulate cortex and hippocampus (37, 38). The amygdala and dorsal anterior cingulate gyrus are essential brain regions for cognitive function and emotional generation and regulation, and enhancing activation will cause hypervigilance in patients (39). Thus, intensive interventions by caregivers targeting the symptom “I was jumpy and easily startled”(H10) “may be more effective in reducing the overall level of PTSD than interventions targeting the remaining symptoms, which may have the greatest clinical significance at the moment.” Psychological interventions can be implemented to reduce the symptoms (40). At present, the common psychological interventions include cognitive behavioral therapy (41), cognitive processing therapy, exposure therapy (42), eye movement desensitization and reprocessing therapy (43). However, it is important to note that each patient’s situation is unique, and it is important to choose treatment options individually. This study provides several important implications for addressing mental disorders in stroke patients. First, the prevalence of PTSD in patients with brain stroke (3-37%) remains alarmingly high (5, 44–48), and our study also found moderate levels of PTSD in stroke patients. This demonstrates the urgent need for timely screening and targeted mental health interventions, the implementation of which is essential to maintain the well-being of stroke patients and maintain quality of life. Second, our study not only provides a detailed understanding of PTSD in stroke patients, but also lays the foundation for future psychological interventions aimed at reducing PTSD in stroke patients. Third, “I was jumpy and easily startled”(H10) is considered to be the core symptom of PTSD in patients with brain stroke. This suggests that in future research, more attention should be paid to whether patients will show negative emotional manifestations after stroke. During health education and nursing intervention, patients should be guided to have a correct understanding of the occurrence of stroke. At the same time, psychological counseling should be provided to patients in a timely manner, encouraging them to express and vent their emotions. Emphasis should be placed on improving patients’ understanding of the disease and eliminating their fears. Reduce the physiological responses caused by fright (49), provide precise rehabilitation guidance, improve the patient’s psychological cognition, avoid emotional imbalance, accelerate the progression of PTSD, and at the same time tell the patient to maintain healthy behaviors and lifestyles. Moreover, in future research, it is necessary to further understand the influencing factors of this symptom, so as to achieve more precise intervention effects, promote the psychological rehabilitation of patients, and more widely reduce the overall level of PTSD.

### Limitations

Current research on NA of PTSD symptoms has primarily focused on populations such as veterans (50) and firefighters (51), while studies examining NA in relation to PTSD among stroke patients remain limited. Our study provides a novel and systematic perspective on the core maintaining factors and potential intervention targets of post-stroke PTSD, thereby offering evidence to support healthcare providers in improving PTSD symptoms in stroke survivors. However, our research still has limitations. First, with the cross-sectional design used in this study, we could not assess PTSD dynamics. A longitudinal investigation of these variables using NA is required. Second, the sample is entirely from the city of Dalian in northern China, so extending the findings to more developed cities remains to be considered. Finally, our study does not empirically confirm the proposed intervention goals and further research is needed to validate them before they can be applied in the real world.

## Conclusion

In summary, this study found the symptom network characteristics and key symptoms of PTSD in stroke patients through symptom network analysis. In future studies, preventive identification of strongly linked and key symptoms should be considered, and scientific psychological intervention measures should be developed to promote the improvement of the mental health status of stroke patients.

## Data Availability

The original contributions presented in the study are included in the article/**Supplementary Material**. Further inquiries can be directed to the corresponding author.
