# Supplementary material for: Network analysis of post-traumatic stress disorder symptoms in stroke patients
Source: Front Psychiatry. 2025 Sep 18;16:1663366. doi: 10.3389/fpsyt.2025.1663366 (PMC12488678; doi:10.3389/fpsyt.2025.1663366)
Supplement: Supplementary file 1 [file DataSheet1.zip › Supplementary material/Table S1. Mean and standard deviation of the PTSD item.docx]

|  | Mean | SD |
| --- | --- | --- |
| I1: Any reminder brought back feelings about it | 1.50 | 0.861 |
| I2: I had trouble staying asleep | 1.71 | 0.918 |
| I3: Other things kept making me think about it | 1.58 | 0.918 |
| H4: I felt irritable and angry | 1.30 | 1.004 |
| A5: I avoided letting myself get upset when I thought about it or was reminded of it | 1.36 | 0.998 |
| I6: I thought about it when I didn't mean to | 1.60 | 0.974 |
| A7: I felt as if it hadn't happened or wasn't real | 1.37 | 0.973 |
| A8: I stayed away from reminders of it | 1.23 | 0.914 |
| I9: Pictures about it popped into my mind | 1.70 | 1.076 |
| H10: I was jumpy and easily startled | 1.62 | 1.205 |
| A11: I tried not to think about it | 1.69 | 1.091 |
| A12: I was aware that I still had a lot of feeling about it, but I didn't deal with them | 1.45 | 0.977 |
| A13: My feelings about it were kind of numb | 1.30 | 1.095 |
| I14: I found myself acting or feeling like I was back at that time | 1.19 | 0.931 |
| H15: I had trouble falling asleep | 1.68 | 1.053 |
| I16: I had waves of strong feelings about it | 1.48 | 1.032 |
| A17: I tried to remove it from my memory | 1.38 | 0.949 |
| H18: I had trouble concentrating | 1.62 | 1.032 |
| H19: Reminders of it caused me to have physical reactions, such as sweating, trouble breathing, nausea, or a pounding heart | 1.09 | 0.896 |
| I20: I had dreams about it | 1.29 | 0.912 |
| H21: I felt watchful and on-guard | 1.33 | 0.990 |
| A22: I tried not to talk about it | 1.57 | 1.177 |
