# Supplementary figures and images for: Network analysis of post-traumatic stress disorder symptoms in stroke patients
Source: Front Psychiatry. 2025 Sep 18;16:1663366. doi: 10.3389/fpsyt.2025.1663366 (PMC12488678; doi:10.3389/fpsyt.2025.1663366)

● Bootstrap mean ● Sample

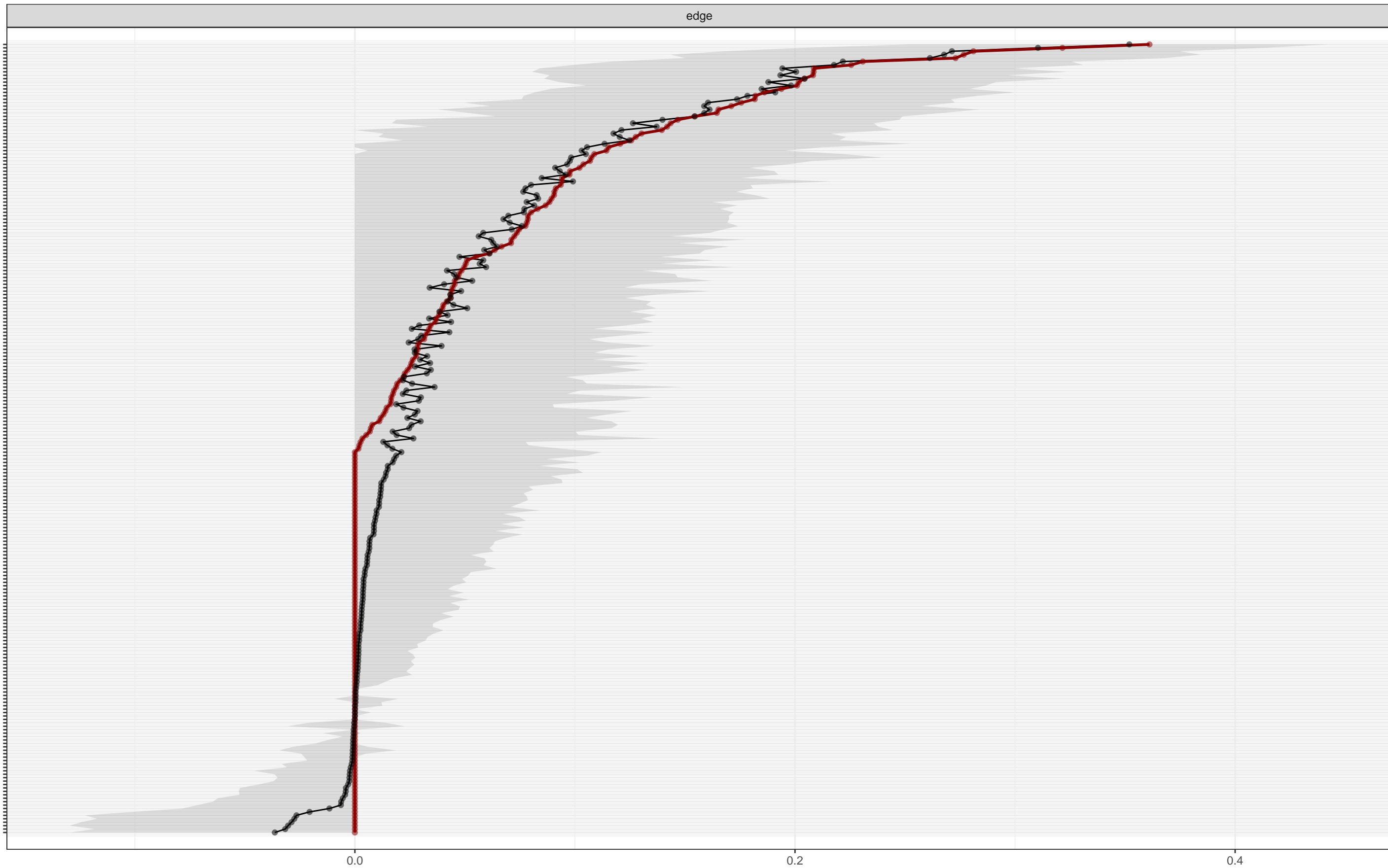

Supplement: Supplementary file 1 [file DataSheet1.zip › Supplementary material/Figure S1.The accuracy of network edges by non-parametric bootstrapping.pdf]

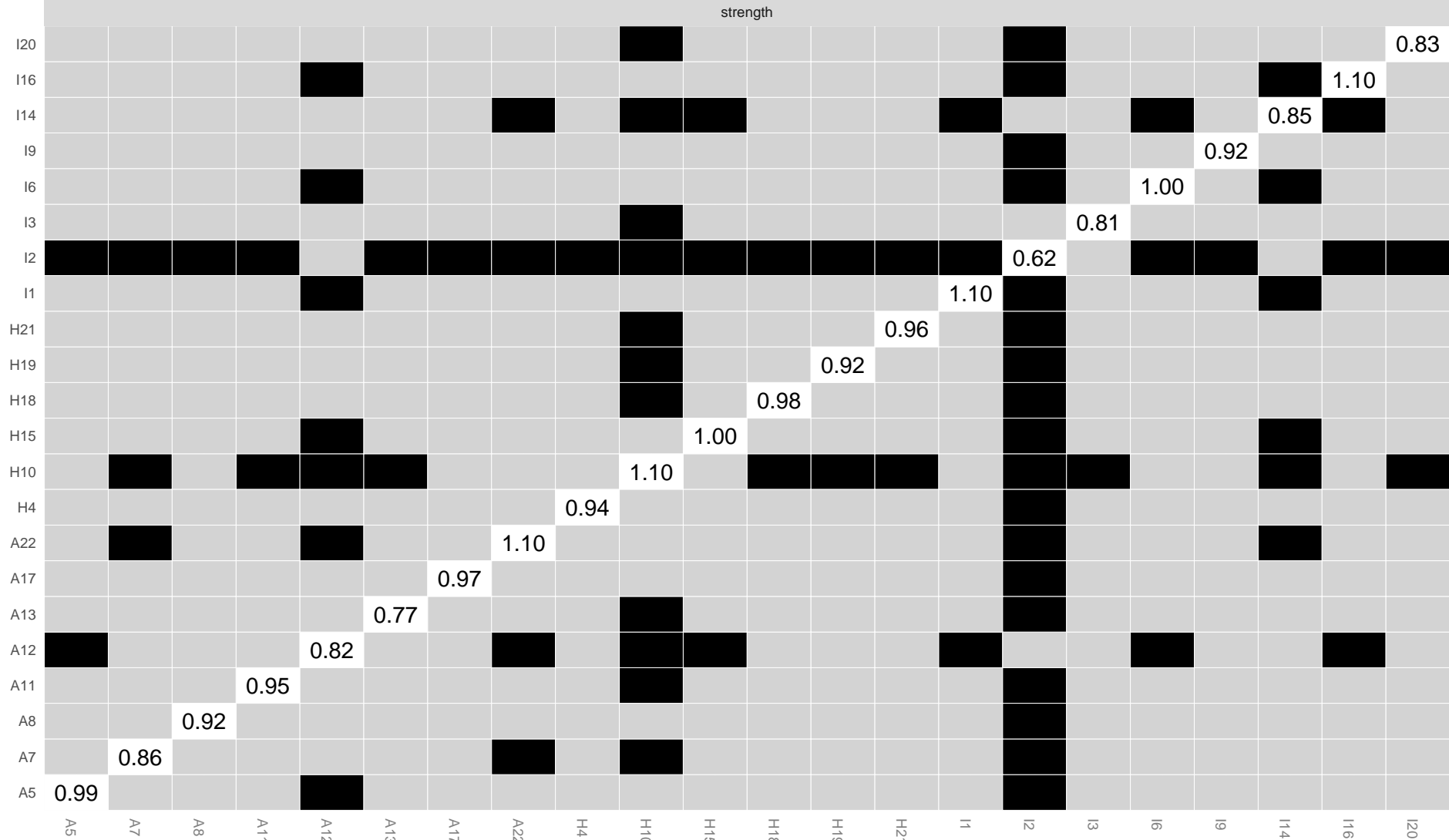

Supplement: Supplementary file 1 [file DataSheet1.zip › Supplementary material/Figure S2. Tests of significant differences between edge weights in the network.pdf]

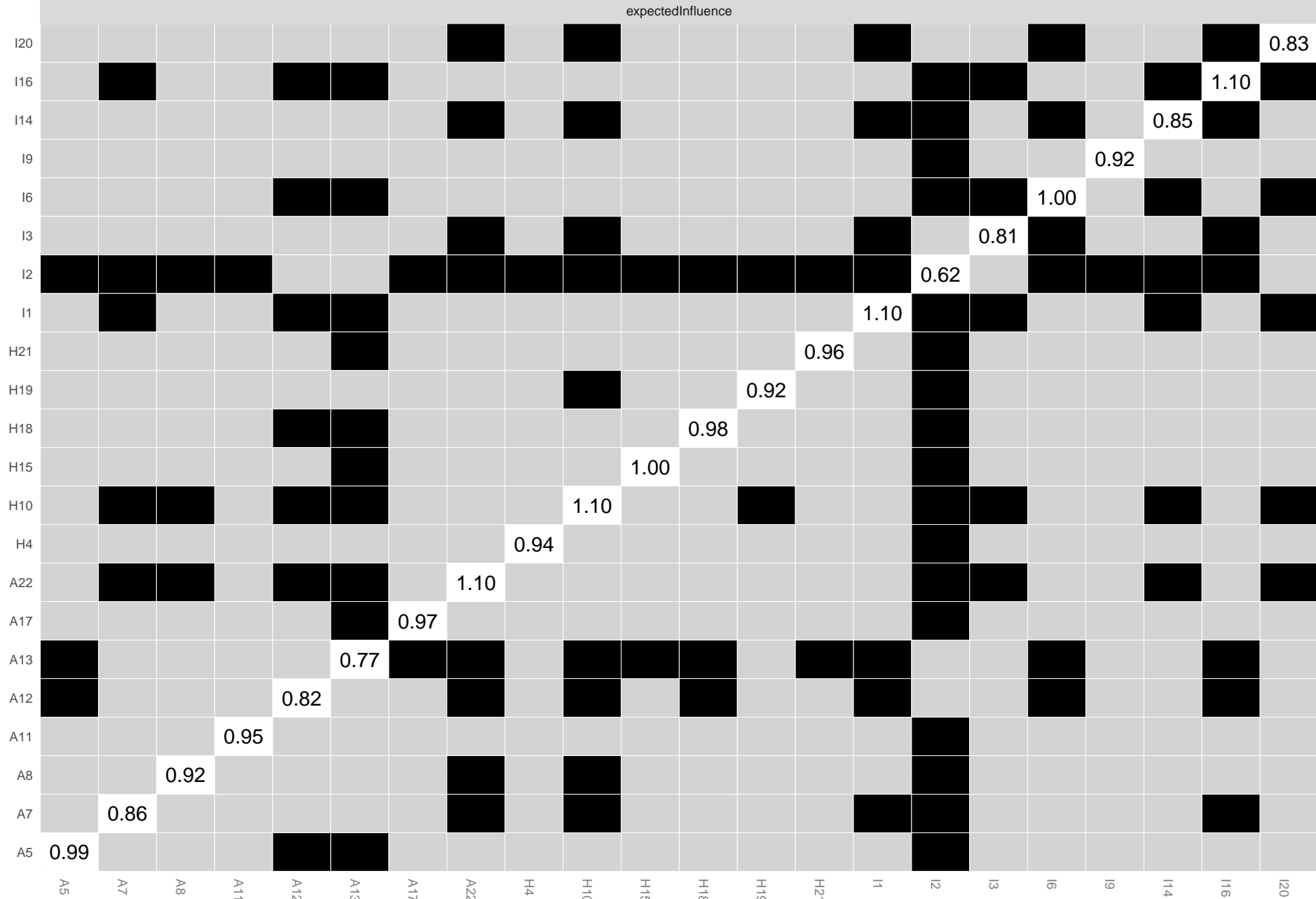

Supplement: Supplementary file 1 [file DataSheet1.zip › Supplementary material/Figure S3. Tests of significant differences between expected influence values ofnodes in the network.pdf]
